# Safe surgical corridor for iliosacral screw placement in unstable pelvic fractures: a computed-tomography-guided validation study of the “triangulation method”

**DOI:** 10.1186/s13037-023-00380-x

**Published:** 2023-11-15

**Authors:** Yu-Bo ZHENG, Xin ZHAO, Qiang ZHENG, Xi-Guang SANG

**Affiliations:** Department of Emergency Surgery and Orthopaedic Surgery, Qilu Hospital of Shandong University, No. 107, West Wenhua Road, Jinan, 250012 Shandong PR China

**Keywords:** Pelvic ring injuries, Iliosacral screw fixation, Triangulation method, Safe surgical corridor, Validation study

## Abstract

**Background:**

The percutaneous iliosacral screw technique represents a global standard fixation method for unstable fractures of the posterior pelvic ring. However, the inaccurate positioning of iliosacral screws is associated with a significant risk of severe intra-operative complications. Therefore, this study aimed to investigate the relationship between the skin entry point of the transverse iliosacral screw of the first sacral vertebral body and the anterior superior iliac spine and the greater trochanter of the femur using computed-tomography-guided validation.

**Methods:**

Overall, 91 consecutive patients admitted to a tertiary referral center in China for posterior pelvic ring fixation via the “triangulation method” using computed-tomography-guided validation between January 1, 2020, and December 31, 2020, were included in this retrospective observational cohort study. Modeling and simulated iliosacral screw placement were performed using the Mimics software. The distance between the three points of interest was measured, and their relationship in a rectangular coordinate system was determined. Patients were categorized according to gender, body mass index, and femoral rotation angle to investigate the factors affecting the positional relationship between the three points.

**Results:**

An equilateral triangular relationship was observed between the positioning points of the transverse iliosacral screw, anterior iliac spine, and greater trochanter. Additionally, 95% of the entry points were within a circle radius centered 12 mm at the apex of an equilateral triangle comprising the anterior superior iliac spine and the greater trochanter as the base. The entry point in the femoral external rotation was more dorsal than that in the internal femoral rotation. Furthermore, the entry point in females was more rostral than that in males, and the entry point in overweight patients was more dorsal than that in normal-weight patients.

**Conclusions:**

The skin entry point of the percutaneous iliosacral screw can be located by drawing an equilateral triangle from the anterior superior iliac spine and the greater trochanter as the base to the dorsum end of the patient’s head. In summary, this retrospective cohort study validated the safety and efficacy of the “triangulation methods” for percutaneous fixation of unstable posterior pelvic ring injuries.

## Background

The percutaneous placement of iliosacral screws is the most common method of posterior ring fixation in unstable pelvic fractures [1–3], with advantages, including short operation time, minimal trauma, less bleeding, low infection rate, and reliable fixation [3–7]. Despite these advantages, precise placement remains challenging because the body’s surface has no obvious exact positioning point during free-hand placement. Achieving a precise placement requires a good understanding of the lumbosacral pelvic region’s anatomy and proficiency in the use of a C-arm [8–10]. Unskilled surgeons may have longer operation and fluoroscopy times, which increases the risk of surgical safety. Furthermore, inaccurate positioning can lead to reduced strength, ineffective screw fixation, or even invasion of the sacral nerve, resulting in pain, numbness, and bladder and bowel incontinence [11, 12].

Several approaches are available for improving the accuracy of iliosacral screw placement, such as robot-assisted and O-arm navigation [13–15]. However, given their high costs and long learning curve, the popularity of robot-assisted and O-arm navigation has waned. Moreover, they are not usually applied promptly to patients with unstable pelvic fractures who require emergency surgery [16]. Rapid and accurate placement of iliosacral screws can quickly control pelvic volume and stabilize the posterior pelvic ring [6]. Therefore, further studies regarding the position of percutaneous iliosacral screw placement to improve the surgical safety of free-hand screw placement are needed.

**Fig. 1:**
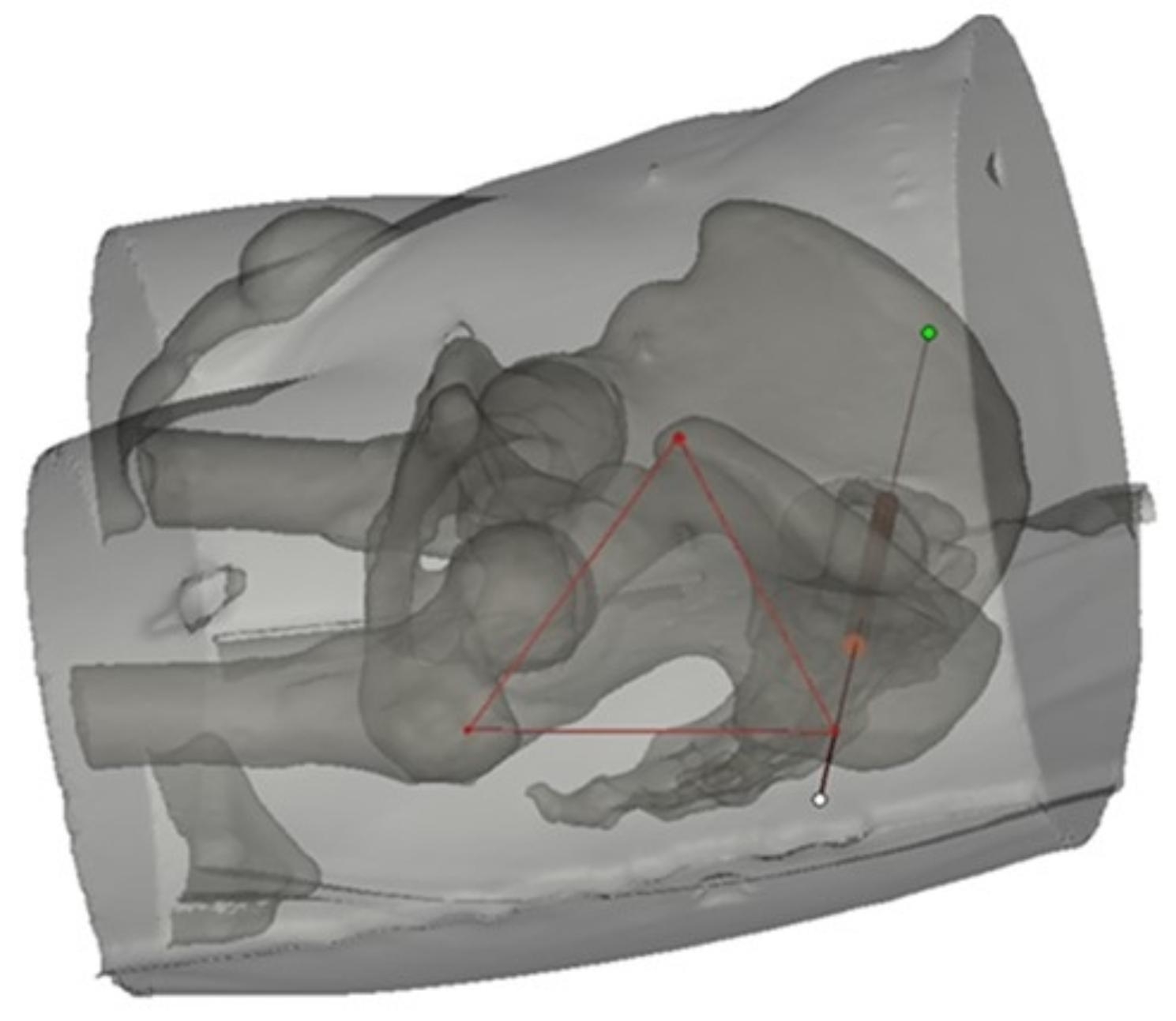
**The positional relationship of the SEP, the ASIS, and the greater trochanter** The skin entry point of percutaneous S1 iliosacral screws showing an equilateral triangle relationship with the ASIS and the greater trochanter ASIS, anterior superior iliac spine; SEP, skin entry point; S1, first sacral vertebral body

Studies about the positioning of iliosacral screws have mainly focused on screw positioning in the ilium and sacrum [2, 8–10, 17]. However, adjusting the position of the guide pin is challenging since the guide device must pass through multiple layers of soft tissue, such as the skin, fascia, and gluteus maximus, prior to reaching the ilium. Therefore, accurate skin positioning during iliosacral screw placement warrants further investigation. We previously observed a fixed geometric relationship between the skin positioning site and the ipsilateral anterior superior iliac spine (ASIS) and the greater trochanter of the femur when the first sacral vertebral body (S1) transverse iliosacral screw was inserted; that is, these three points appear to form an equilateral triangle (Fig. 1). Therefore, this study aimed to determine the optimal skin entry point using three-dimensional (3D) modeling to simulate the placement of iliosacral screws and the positional relationship between the skin entry point, the ASIS, and the greater trochanter.

## Methods

### Patient selection

This is a retrospective, cross-sectional analysis of the patients in Qilu Hospital. Computed tomography (CT) data of the pelvis of patients who visited our hospital between January 1, 2020, and December 31, 2020, were collected. The inclusion criteria were (i) patients aged 18–60 years and (ii) those with CT scans of the complete pelvis and the greater trochanter of the femur. Exclusion criteria included patients with (i) a fracture present on a CT image, (ii) hip arthritis, (iii) pelvic deformity or osteolytic disease such as a bone tumor, and (iv) a 7.3-mm screw difficult to be placed transversely to the S1.

### Processing and reconstruction of CT data

Anonymized CT raw data were post-processed using the Mimics 25.0 software (Mimics, Materialise N.V., Belgium). The appropriate range of Hounsfield units (HU) was selected to locate the boundaries of the cortical bone or soft tissue during reconstruction. The 3D reconstruction function was used to construct a 3D model of bone tissue, fill the space of the bone tissue samples after construction, and smooth the process. The soft tissue model, in this case, was constructed using a similar method.

### Surgical intervention

**Fig. 2:**
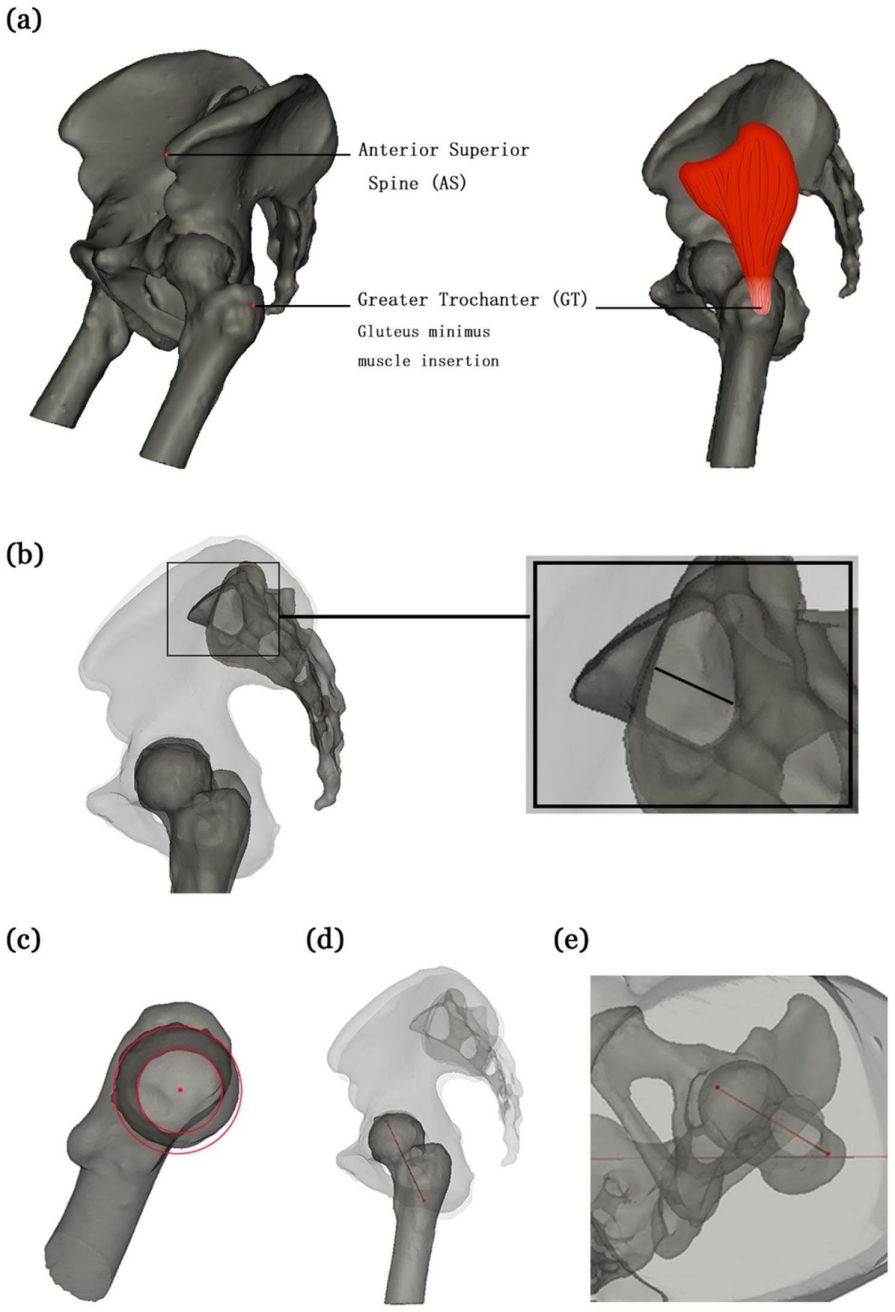
**Measurement of anatomical data** (**a**) The ASIS and the greater trochanter position, (**b**) safe access for iliosacral screws, (**c**) concentric circles of the femoral head and neck, (**d**) the femoral neck axis, and (**e**) the femoral rotation angle ASIS, anterior superior iliac spine

The ipsilateral ASIS and the greater trochanter were marked. The ASIS was selected as the highest point of the pelvis in the sagittal position in a supine position (Fig. 2a). The greater trochanter was marked at the center of the gluteus minimus insertion in the middle of the plane of the most lateral vertex of the greater trochanter toward the cephalic end (Fig. 2a). This point was selected because it is more accurate to touch the plane of the greater trochanter, where the gluteus minimus points during actual surgery.

To simulate the placement of iliosacral screws, the bone tissue was rendered semitransparent (Fig. 2b), and the 3D model was adjusted to the standard lateral view (bilateral ASIS and alignment of the high-density line). The shadow formed by the first sacral pedicle, which was roughly oval, was visible. The upper and lower limits of the ellipse were the sacral alar, and the posterior and lower boundaries indicated the sacral nerve canal. The length of the short axis of the shadow was measured. Additionally, the short shaft was at least > 7.3 mm in length. Samples with short axis lengths < 7.3 mm were excluded. The midpoint of the short axis was the best position for the placement of iliosacral screws. The two ends of the simulated screw were extended, and the intersection point between the extension line and the skin was the skin entry point. Furthermore, the linear distances between the skin entry point, the greater trochanter, and the ASIS were measured.

### Measurement of the angle of the femoral neck axis and the coronal plane

In the 3D reconstruction model of bone tissue, the view angle was adjusted such that the unilateral femoral head and femoral neck were observed as concentric circles (Fig. 2c), and the center of the concentric circle from the inner and outer sides of the femoral neck was marked (Fig. 2d). The inner and outer sides of the concentric circle center were connected by a line defined as the femoral neck axis. Furthermore, the angle of the femoral neck axis between the horizontal projection and coronal plane was measured (Fig. 2e) and defined as the femoral rotation angle.

### Data processing and rectangular plane coordinate system

To compensate for individual differences, the distance between the ASIS and the greater trochanter of the femur was defined as standard length 1, and the distance between the simulated needle entry point and the ASIS and the greater trochanter of the femur was standardized.

**Fig. 3:**
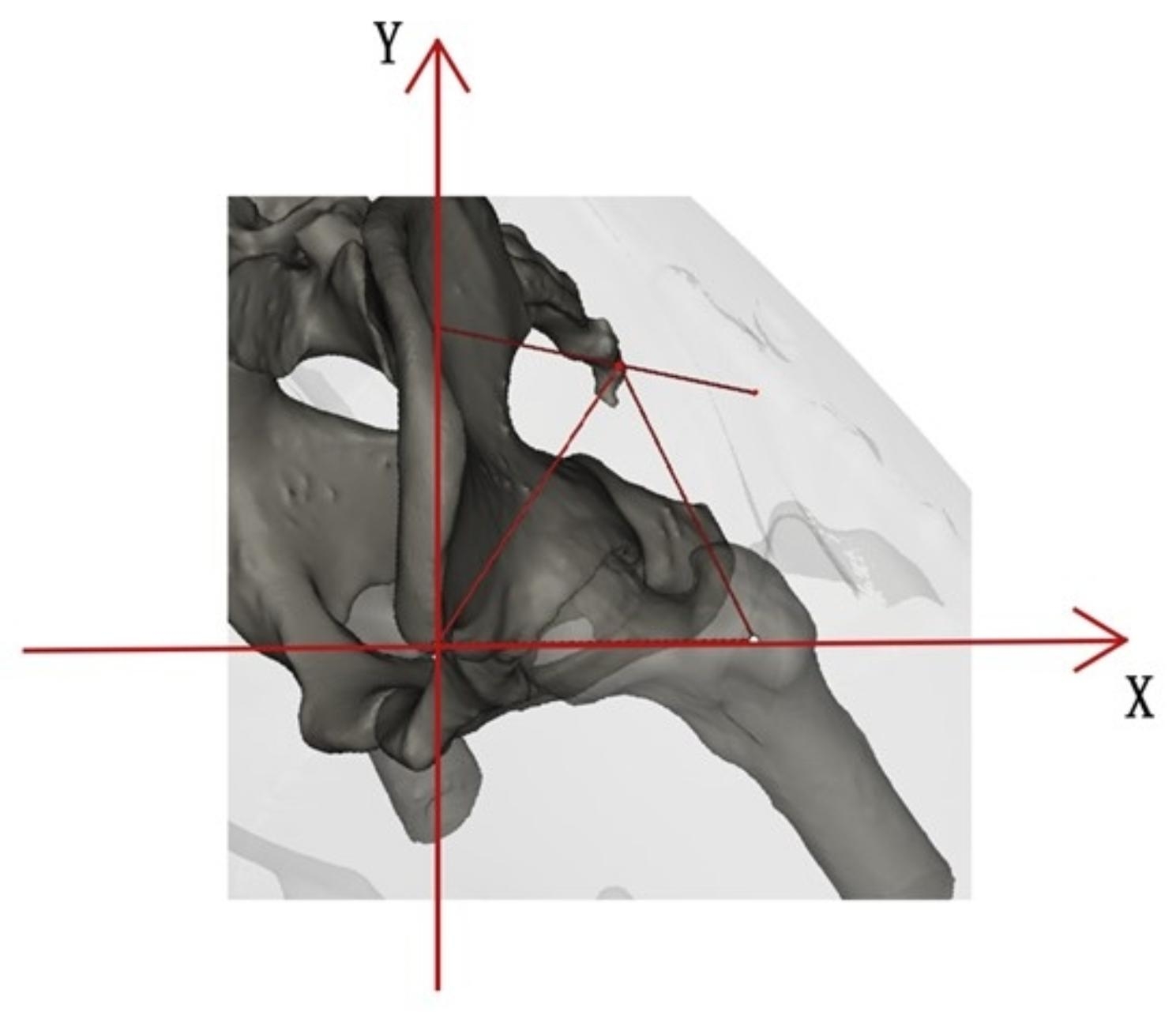
**Rectangular plane coordinate system** The plane cartesian coordinate system is established in the plane where the three points, namely, the greater trochanter, the ASIS, and the SEP, are located ASIS, anterior superior iliac spine; SEP, skin entry point

On the plane determined using the ASIS, the greater trochanter, and the skin entry point, the ASIS was used as the origin, the ASIS to the greater trochanter of the femur was used as the X-axis, and the injection point was simulated as the positive side of the Y-axis to establish a rectangular plane coordinate system (Fig. 3). The distance between the simulated needle entry point and the ipsilateral ASIS and the greater trochanter of the femur was measured. Trigonometric functions were used to calculate the position of the simulated entry point of the needle using X- and Y-coordinates, which were statistically analyzed. This operation yielded 182 scatter points, and normality tests were performed using the X- and Y-coordinates. The point in the plane of the coordinate system with the shortest distance from all scatter points was defined as the average point to calculate the median center, as follows:

$$f\left(x\right)=\sum\limits_{i=n}^n\sqrt{{ ({x}_{n}-a)}^{2}+{ ({y}_{n}-b)}^{2}}$$

When the function takes its minimum value, (a, b) is the average point of the scatter. This operation was performed using Python, version 3.9 (Python Software Foundation, Delaware, USA) software.

We used kernel density estimation, which is a non-parametric probability density estimation method, for calculating the aggregation of scatter points in the plane, and we constructed a density distribution map using Python version 3.9 software (Python Software Foundation, Delaware, USA).

### Statistical analysis

A Shapiro–Wilk test was used to determine the normal distribution of quantitative data. Student’s *t*-test or Welch’s nonparametric test was used to compare the means or data distributions between the different groups. Statistical significance was set at P < 0.05. All statistical analyses were performed using IBM SPSS Statistics for Windows, version 26.0 (IBM Corp., Armonk, N.Y., USA).

## Results

### Patient demographics

In this study, 91 CT scans were collected, and both left and right sides were measured, resulting in 182 sets of data. Table 1 presents the baseline values.

### Operating points and factors influencing penetration parameters of entry points

**Fig. 4:**
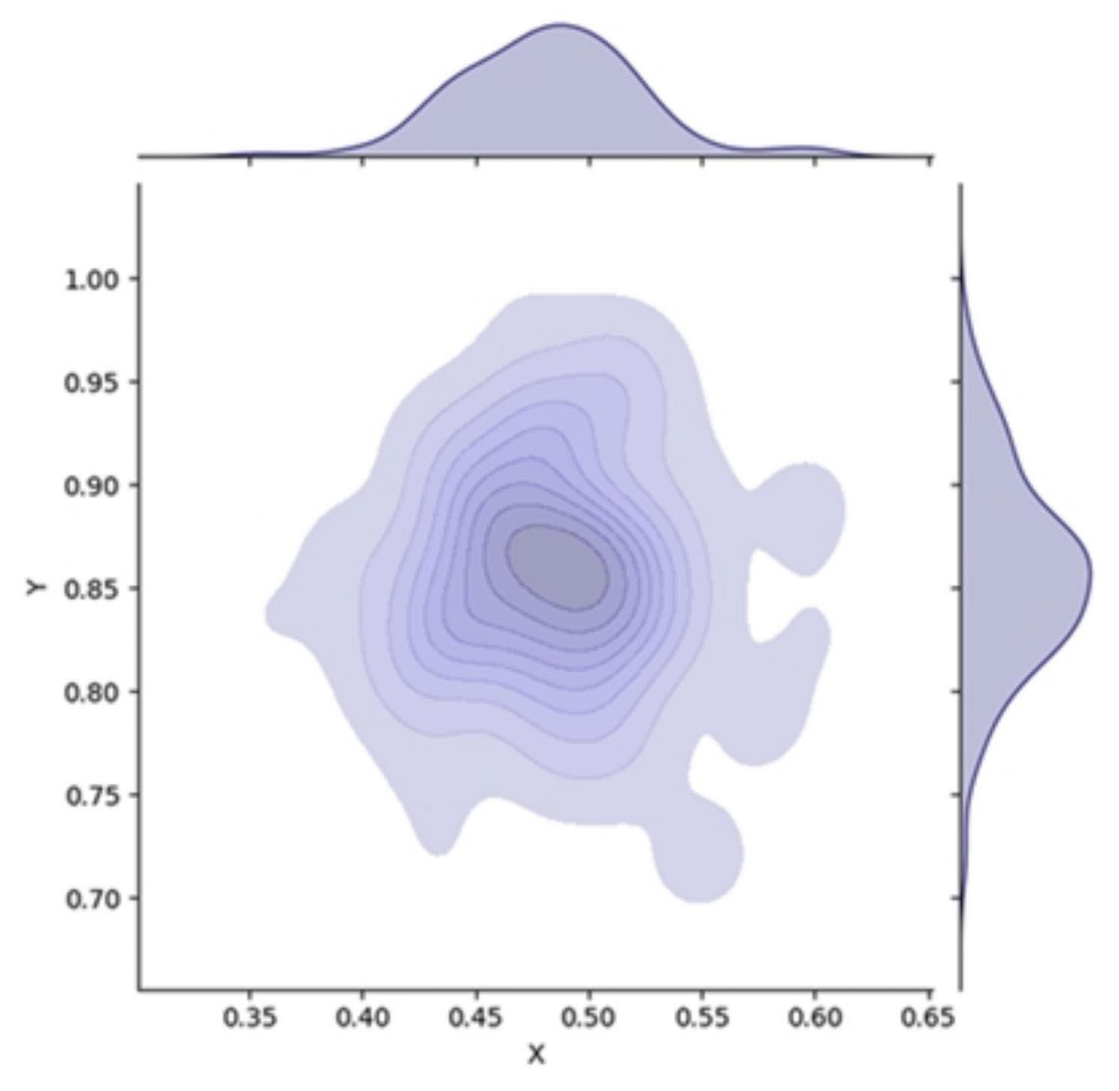
**Plot of kernel density estimation for overall SEPs** SEP, skin entry point

The average point was (0.49, 0.86). Figure 4a shows the probability distribution density. The abscissa and ordinates of the operation points were normally distributed; accordingly, each point had a two-dimensional normal distribution in the plane. The average distance between each needle entry point and the average point was 0.0566 (0.0512–0.0619), and the predicted point (the vertices of the regular triangle) was (0.50, 0.87). The predicted point was 0.01 units away from the average point, and the actual average distance was 1.15 mm. Approximately 95% of the entry points were within a circle radius of 12 mm (0.50, 0.87).

A one-sample *t*-test was performed on the abscissa and ordinate of the vertex of the regular triangle (0.5, 0.87) and the abscissa and ordinate of the entry point set. In the coordinate system, the average value of the receiving needle entry point’s abscissa and the ordinate was 0.5 (P < 0.001) and 0.87 (P < 0.001), respectively. Accordingly, the skin entry point of the S1 transverse penetration iliosacral screw was considered to have a regular triangular relationship with the ASIS and the greater trochanter.

**Table 1:** Patient demographics

Variables	
Age (mean ± SD, years)	42.92 ± 9.08
Gender (male/female)	88/94
Height (mean ± SD, m)	1.68 ± 0.08
Weight (mean ± SD, kg)	68.64 ± 13.73
BMI (mean ± SD, kg/m^2^)	24.17 ± 2.96
Femoral rotation angle (mean ± SD, °)	24.83 ± 5.72
ASIS–greater trochanter distance (mean ± SD, mm)	115.24 ± 13.00
ASIS–skin entry point distance (mean ± SD, mm)	113.51 ± 13.79
Greater trochanter–skin entry point distance (mean ± SD, mm)	115.46 ± 12.88
Abscissa (mean ± SD)	0.48 ± 0.040
Ordinate (mean ± SD)	0.86 ± 0.049

ASIS, anterior superior iliac spine; BMI, body mass index; SD, standard deviation

### Femoral rotation angle

The greater trochanter is separated from the surgical area through the hip joint; therefore, the femoral rotation angle may influence the accurate positioning of the needle entry point. When a normal human body is relaxed and lying supine, the hip joint is affected by gravity, and external rotation occurs. Therefore, categorizing patients according to their femoral rotation angle and investigating the influence of hip joint position on positioning is necessary.

**Fig. 5:**
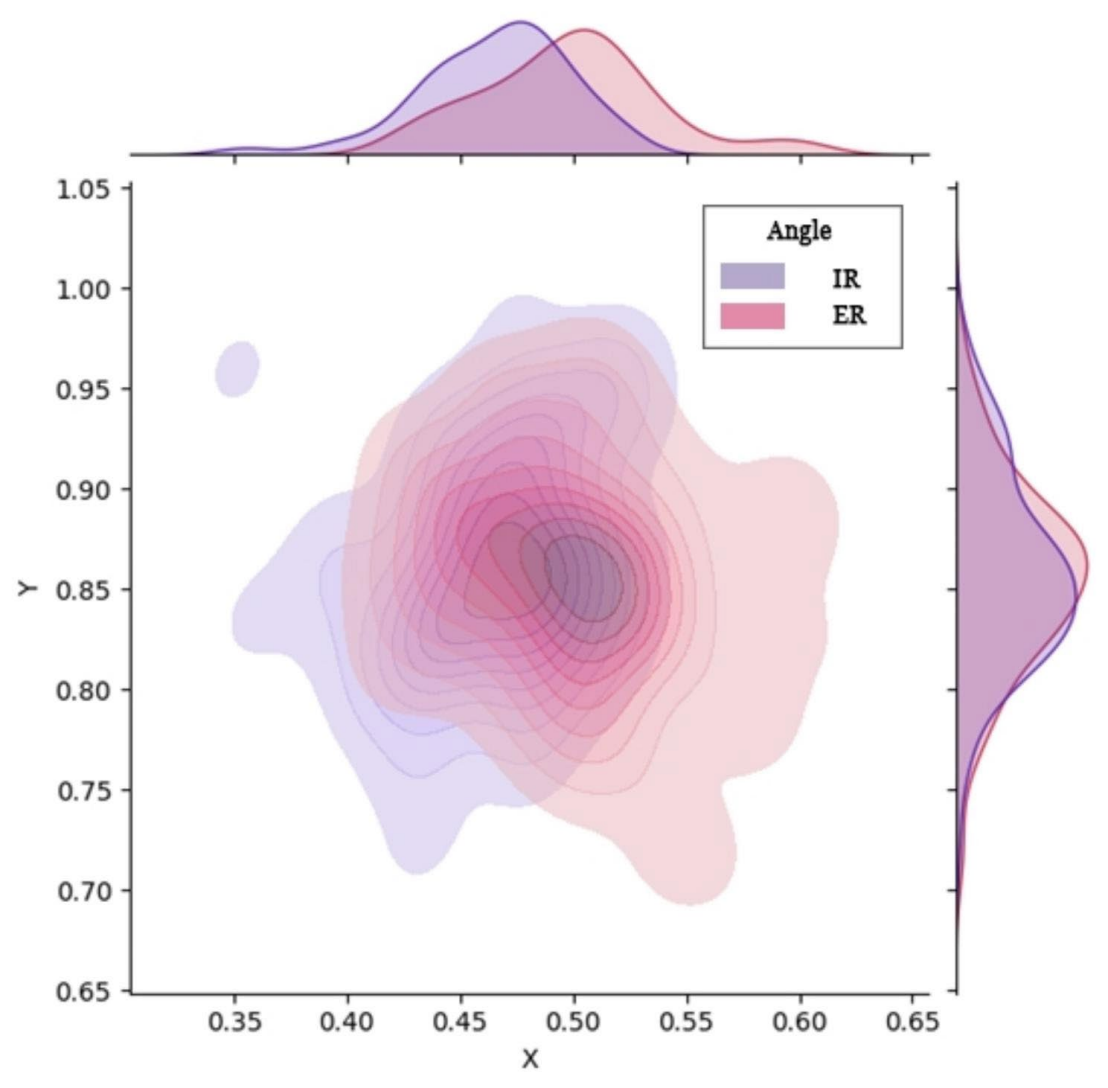
**Kernel density distribution plots grouped according to femoral rotation angle** IR, internal rotation; ER, external rotation

The median measured rotation angle was 25.20° (22.20°–27.10°), and the anteversion angle of the femoral neck ranged from 12° to 15°. Considering the inevitable mild femoral external rotation due to the effect of gravity under anesthesia, the patients were classified into internal (≤ 25°) and external (> 25°) rotation groups according to the distribution of measurement angles. The X- and Y-coordinates for each group of data were normally distributed. The probability density plot (Fig. 5) shows clear differences between the two groups. Two independent sample *t*-tests were used to compare the two groups, and significant differences were observed between the abscissae (P < 0.001). No significant differences were found between the ordinates (Table 2). The skin entry point of the internal and external rotation groups was inclined to the ventral and dorsal sides, respectively.

**Table 2:** Grouping according to femoral rotation angle

Variables	Internal rotation group (n = 88)	External rotation group (n = 94)	Effect size	P-value
Gender (male/female)	42/46	46/48	*χ*^2^ = 0.027	0.870
BMI (mean ± SD, kg/m^2^)	23.71 ± 2.70	24.64 ± 3.13	T = 2.073	0.04
Abscissa (mean ± SD)	0.46 ± 0.033	0.50 ± 0.039	T = 6.309	< 0.001
Ordinate (mean ± SD)	0.86 ± 0.049	0.86 ± 0.048	T = 0.623	0.534

BMI, body mass index; SD, standard deviation

### Gender

**Fig. 6:**
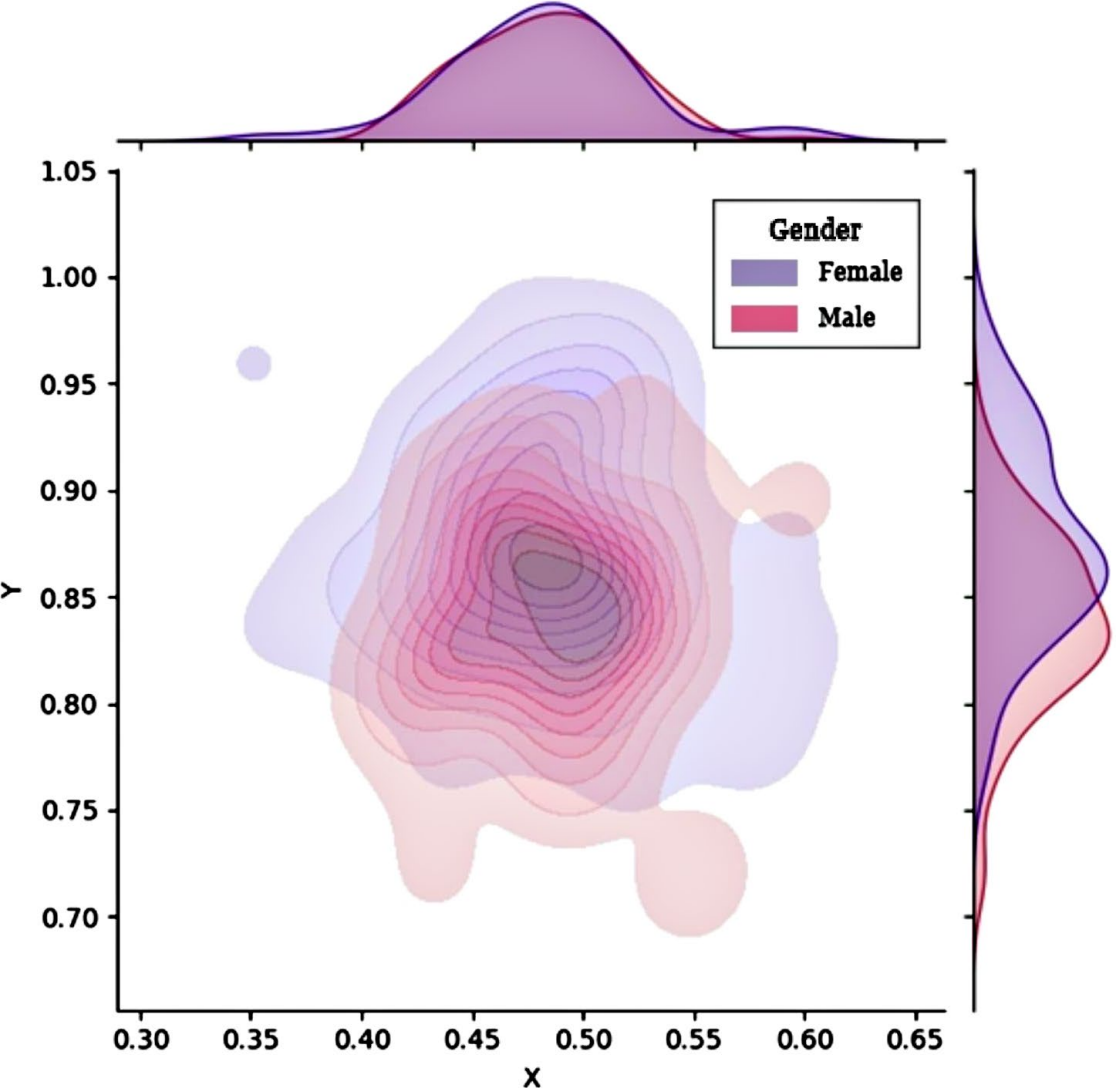
Kernel density distribution plots grouped according to gender

Gender may be an influencing factor because of the differences in pelvic structure between males and females; therefore, patients were categorized according to gender. Figure 6 shows a probability density curve. The abscissae and ordinates in the two groups were tested using two independent sample *t*-tests. A significant difference was observed between the ordinates but not between the abscissae (P < 0.001, Table 3). The mean point position was more cephalic in males than in females.

**Table 3:** Grouping according to gender

Variables	F (n = 94)	M (n = 88)	Effect size (T)	P-value
BMI (mean ± SD, kg/m^2^)	24.82 ± 2.59	23.49 ± 3.12	3.088	0.002
Femoral rotation angle (mean ± SD, °)	24.82 ± 5.43	24.82 ± 6.06	0.002	0.999
Abscissa (mean ± SD)	0.48 ± 0.044	0.48 ± 0.036	0.336	0.738
Ordinate (mean ± SD)	0.88 ± 0.047	0.83 ± 0.043	5.670	< 0.001

BMI, body mass index; F, female group; M, male group; SD, standard deviation

### Body mass index

**Fig. 7:**
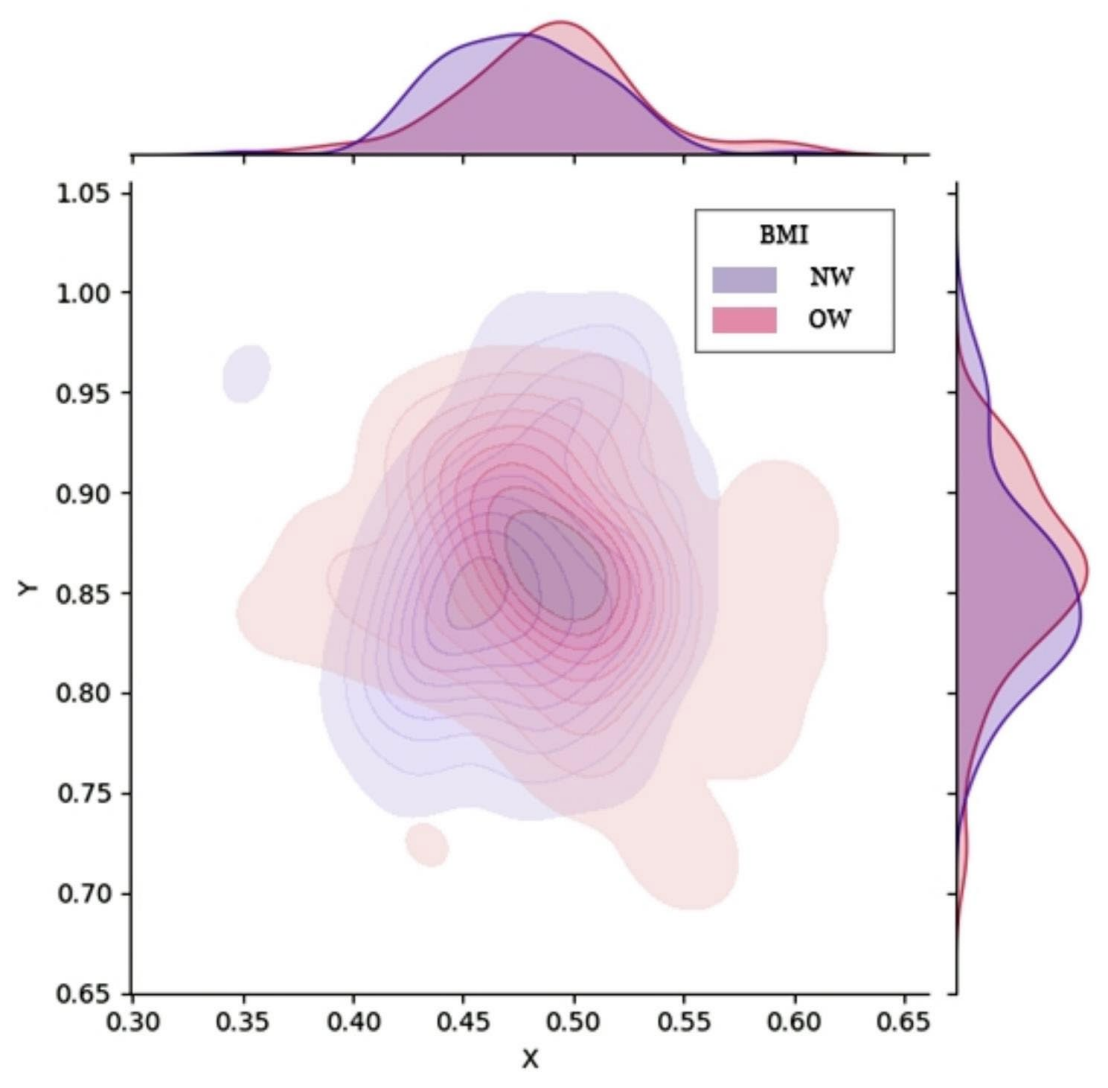
**Kernel density distribution plots grouped according to BMI** BMI, body mass index; NW, normal-weight; OW, overweight

Body mass index (BMI) may be an influencing factor because of the traversal soft tissue. The mean patient BMI was 23.84 ± 2.88 kg/m^2^. Patients were classified into the normal-weight (BMI ≤ 26 kg/m^2^) and overweight (BMI > 26 kg/m^2^) groups. Figure 7 shows the probability density plots. Two independent sample *t*-tests were performed on the abscissae and ordinates. Significant differences were observed in the abscissae (P = 0.020) but not in the ordinates (Table 4). The normal-weight group was more inclined to the ventral side of the body, whereas the overweight group was more inclined to the dorsal side.

**Table 4:** Grouping according to BMI

Variables	N (n = 132)	OW (n = 50)	Effect size	P-value
Gender (male/female)	68/64	20/30	*χ*^2^ = 1.926	0.165
Femoral rotation angle (mean ± SD, °)	24.42 ± 5.61	25.87 ± 5.95	T = 1.491	0.140
Abscissa (mean ± SD)	0.48 ± 0.038	0.49 ± 0.043	T = 2.346	0.020
Ordinate (mean ± SD)	0.86 ± 0.048	0.86 ± 0.051	T = 0.011	0.991

BMI, body mass index; N, normal-weight group; OW, overweight group; SD, standard deviation

## Discussion

This study aimed to provide a precise entry point for the manual placement of a percutaneous transverse-penetrating iliosacral screw. Iliosacral screws can be broadly classified into two types based on their angle of insertion. The first is the oblique screw placement method [4]. The oblique iliosacral screw is placed in the direction of the sacral wing, and the caudal end of the screw is oblique to the dorsal and caudal ends of the human body. Therefore, only one side of a sacral fracture or iliosacral joint dislocation can be fixed. The second type is transverse insertion [18]. The transverse placement of iliosacral screws is perpendicular to the sagittal plane of the human body and generally penetrates the bilateral iliosacral joints to enable these screws to penetrate six layers of the cortical bone, which provides higher stability [19]. Furthermore, the transverse placement of iliosacral screws can stabilize the bilateral iliosacral joints and fix the median sacral fracture zone 3 (Denis 3). Trans-sacral screws can be applied to almost all sacral fractures or dislocations of the iliosacral joint and have better fixation effects, except in some patients who do not have a safe transverse penetration placement channel. However, the sacrum’s internal anatomy is more complex since the nerves of the lumbosacral trunk pass through the sacral wings on both sides of the sacral promontorium, and the sacral nerve canal passes through the sacral wings. Therefore, the iliosacral screw has more stringent positioning requirements.

Many studies have evaluated the entry point of iliosacral screws into the sacrum. Mendel et al. proposed that the entry point of iliosacral screws should be determined using lateral pelvic radiography to observe safe passage through the iliosacral region [8]. Gusic suggested that the average entrance view of the first sacral passage and the average exit view should be 22.3° (range, 10.4°–39.8°) and 42.3° (31.5°–53.1°), respectively, to better observe the angle of screw deviation in the sacrum [9]. Ozmeric considered that it was necessary to observe the entrance position of the first sacral passage from two different angles to reduce misjudgment [10]. Most studies have focused on positioning the screw in the ilium and sacrum after traversing the skin and other soft tissues. However, iliosacral screws pass through tough connective tissue, such as the gluteus maximus, during the placement process. Since erroneous positioning on the body surface may easily occur, adjusting the screw or the guide pin under the skin, muscle, or ligaments would be difficult. Even if adjustments were forcibly achieved, the deviation would need to be monitored using the C-arm during the insertion process. Therefore, determining an accurate positioning point on the skin is important.

Iorio et al. used a four-zone method to locate the entry point of iliosacral screws [20]. However, this method only describes a general direction and requires several adjustments during the operation. Furthermore, this conventional method completely depends on better trochanter positioning; the hip joint is located between the needle entry point of the surgical position and the greater trochanter, which causes a deviation in positioning due to the hip joint’s rotation. As a pestle and acetabular joint, the hip joint’s range of motion is second only to that of the shoulder joint. The angle of femoral external rotation also affects positioning.

The S1 transverse iliosacral screw was found to have a fixed positional relationship with the ipsilateral ASIS and the greater trochanter of the femur; that is, the three constitute an equilateral triangle. In this study, the CT pelvic data of 91 patients were collected, and the transverse placement of penetration screws was simulated. The intersection point between the straight line of the screw and the skin was the best surgical skin positioning point. Distances between the three points were measured, and coordinates were determined. The equilateral triangular relationship between the three points was verified using statistical methods. Of the skin entry points, 95% were within a circle radius centered 12 mm at the apex of an equilateral triangle comprising the ASIS and the greater trochanter as the base.

However, the skin entry point of the S1 transverse iliosacral screw needs to be adjusted according to each specific situation. A femur is not always placed in a neutral position under anesthesia, and external rotation of the femur may shift the entry point to a more dorsal position. Therefore, when the surgeon observes that a patient is lying with their hips fully externally rotated, the skin entry point should be adjusted accordingly. Additionally, since the pelvis is long and narrow in males and wide and flat in females, the triangulation method would differ according to gender. The skin entry point was more cephalic in males than in females. In patients who were overweight or obese, the distance between the skin entry point and bony marker increased due to excessive subcutaneous fat, and the anchor point’s position was more cephalic. Moreover, significantly thick subcutaneous fat will also require a surgeon to fully evaluate the position of the greater trochanter. Therefore, the triangulation method is not as accurate for overweight patients compared with normal-weight patients.

## Conclusions

Overall, the skin entry point for transverse S1 iliosacral screw placement can be easily located using the percutaneous iliosacral screw technique proposed in this study. In total, 95% of the entry points were within a circle radius centered 12 mm at the apex of an equilateral triangle comprising the ASIS and the greater trochanter as the base. Additionally, femoral rotation angle, gender, and BMI affected this positioning method.

## Data Availability

The datasets generated and analyzed during the current study are available from the corresponding author upon reasonable request.

## List of abbreviations

ASIS: anterior superior iliac spine
BMI: body mass index
CT: computed tomography
SD: standard deviation
S1: first sacral vertebral body
